# Association between serotonin 2C receptor gene (*HTR2C*) polymorphisms and psychopathological symptoms in children and adolescents

**DOI:** 10.1590/1414-431X20187252

**Published:** 2018-06-14

**Authors:** L.A. Paes, O.H. Della Torre, T.B. Henriques, M.P. de Mello, E.H.R.V. Celeri, P. Dalgalarrondo, G. Guerra, A. dos Santos

**Affiliations:** 1 Departamento de Psicologia Médica e Psiquiatria, Universidade Estadual de Campinas, Campinas, SP, Brasil; 2 Centro de Biologia Molecular e Engenharia Genética, Universidade Estadual de Campinas, Campinas, SP, Brasil; 3 Departamento de Pediatria, Universidade Estadual de Campinas, Campinas, SP, Brasil

**Keywords:** Genetic polymorphism, Serotonin, CBCL/6-18, Children, Adolescents, HTR2C

## Abstract

Serotonin 2C receptors (5HT2C) are involved in serotonin-driven dynamic equilibrium adjustments responsible for homeostatic stability in brain structures that modulate behavior and emotions. Single nucleotide polymorphisms (SNPs) from the serotonin 2C receptor gene (*HTR2C*) have been associated with several neurological and mental disorders, including abnormalities in cognitive and emotional processes. The aim of this study was to evaluate the association between the rs6318 SNP of the *HTR2C* gene and behavioral characteristics exhibited by children and adolescents based on the Child Behavior Checklist (CBCL/6-18) inventory. Eighty-five psychiatric outpatients between 8 and 18 years of age underwent genotyping of the rs6318 SNP. The CBCL/6-18 scale was administered to their caregivers. The chi-squared test was used to assess differences in the frequency of C and G alleles of the rs6318 SNP relative to the grouped CBCL/6-18 scores; significance level was 5%. The presence of the G allele of rs6318 was found to be associated with characteristics of aggressive behavior and social problems, and aggressive behavior was found to be associated with heterozygosis in females. These findings contribute to the identification of mental and behavioral phenotypes associated with gene expression.

## Introduction

Mental health issues among children and adolescents are relevant because they are common, usually persist into adulthood, and impact other individuals and society at large (1). In Brazil, the overall prevalence of one or more psychiatric disorder among school-age children and adolescents is approximately 13.1%, a rate similar to averages found worldwide (2).

Highly effective instruments can predict symptom patterns in children and adolescents. The Child Behavior Checklist (CBCL/6-18) is a parental rating inventory based on the fourth edition of the Diagnostic and Statistical Manual of Mental Disorders (DSM-IV) (3), and it is widely used in scientific studies (4,5). Bordin et al. (6,7) have translated and validated the CBCL/6-18 inventory for the Brazilian population.

Empirical studies report that psychopathological traits are strongly associated with genetics as early as childhood, and there have been attempts to identify possible determinants on the molecular level, at which specific polymorphisms and alleles may be associated (8,9). A variation at a single position in the DNA sequence is defined as a single nucleotide polymorphism (SNP) if more than 1% of a population carries that variation. The alteration can lead to variations in the amino acid sequence (10,11). Some SNPs have been described as being associated with psychiatric disorders (12).

The serotonin 2C receptor gene (*HTR2C*), located at Xq23 (13), encodes for the serotonin 2C receptor (5HT-2C). This receptor is involved in moment-to-moment homeostatic regulation of the excitatory-inhibitory balance of neurophysiological processes, and it has been found to aid in homeostatic stability and the prevention of allostatic overload (14). Homeostasis is understood as the interactions between automatic and constant physiological processes that promote health and well-being. In contrast, allostatic regulations shift these operating ranges when the body encounters new challenges, rewards or threats that require an active coping response (15,16). Autoradiographic studies have identified 5HT-2C in the choroid plexus, cerebral cortex, nucleus accumbens, hippocampus, ventromedial prefrontal cortex, and amygdala (17).

There are 911 known SNPs within the *HTR2C* gene, and 390 have been validated. These 390 SNPs are good candidates for association studies (14). The rs6318 (C/G) SNP, in particular, is a frequent mutation in nucleotide 68 associated with a cysteine (G allele) to serine (C allele) substitution (forward direction) at amino acid 23 of the protein sequence. This substitution could disrupt a disulfide bridge (14).

The objective of this study was to determine possible associations between the rs6318 (C/G) SNP and behavioral symptoms among children and adolescents receiving outpatient psychiatric care.

## Material and Methods

This was a descriptive cross-sectional study. The subjects were children and adolescents who had participated in a previous study that quantified the incidence of obesity, hypertension, insulin resistance, metabolic syndrome, and hyperprolactinemia in children and adolescents to whom risperidone was administered to treat mental and behavioral disorders (18–20). The inclusion criteria allowed for children and adolescents between the ages of 8 and 18 who were treated in the outpatient psychiatric unit of the Universidade Estadual de Campinas (UNICAMP) Hospital das Clínicas, São Paulo, Brazil between March 2014 and August 2015. This clinic is designed for severe mental health cases requiring tertiary care. This public hospital receives referrals from more than 100 cities in the state of São Paulo (21). The exclusion criteria were prior obesity, the use of medication known to cause metabolic syndrome, physical illness that could alter metabolic parameters, regular use of psychoactive drugs, moderate or severe intellectual disability, and diagnosis of an eating disorder.

All of the subjects from the previous study were invited to participate in the current study. Patients and their guardians provided informed consent before entering the second study (UNICAMP Research Ethics Committee Form 44199; Certificate of Presentation for Ethical Consideration [CAAE] Registry No. 04369612.8.0000.5404; June 26, 2012).

The psychiatric diagnoses of the patients were based on the International Statistical Classification of Diseases (ICD-10) (22), and this information was obtained from their medical records. Forty-two subjects (49.4%) had conduct disorders (F91) or mixed disorders of conduct and emotions (F92); 35 (41.2%) had hyperkinetic disorders (F90); 29 (34.1%) had depressive disorders (F32, F33, F34, F38, and F39); 24 (28.2%) had mild mental retardation (F70); 20 (23.5%) had pervasive developmental disorders (F84); 17 (20%) had neurotic, stress-related, or somatoform disorders; and 6 (7.1%) had schizophrenia (F20) or schizotypal disorders (F21). Information on the use of psychiatric drugs was also obtained from their medical records. All of the patients were undergoing treatment with risperidone, which had been given for a mean of 34.6±23.5 months at the time of data collection. Nineteen patients (22.7%) were receiving the medication as monotherapy, 48 (54.1%) were also receiving antidepressants, 23 (27.1%) were receiving risperidone associated with a psychostimulant, and 11 (12.9%) were receiving risperidone associated with clonidine. Fifteen patients (17.6%) were receiving other associated drugs, which included anticonvulsants, lithium, benzodiazepines, and other antipsychotic drugs.

The CBCL/6-18 scale was administered to the patients' parents or guardians. This instrument tracks behavioral changes in children and adolescents over the six months prior to its administration. It was applied after each patient's scheduled psychiatric appointment.

The inventory is composed of 138 questions answered on a Likert scale, 20 of which refer to a competence scale score and 118 address behavioral problems (7). Respondents must assign one of the following scores to the problems addressed in each question: 0 if it is not true; 1 if it is somewhat or sometimes true, or 2 if it is very true or often true. For the calculation of the final score, the Assessment Data Manager (ADM) software (ASEBA¯ Achenbach System of Empirically Based Assessment, USA) was used. The software provides T-scores, a chart analysis, and a report.

The competence scale score is calculated as the sum of the raw scores of the activities, social, and school subscales. The behavioral problems are separated into three groups: syndrome scales (anxious/depressed, withdrawn/depressed, somatic complaints, social problems, thought problems, attention problems, rule-breaking behavior, and aggressive behavior); broadband scales (internalizing problems, externalizing problems, and total problems, the latter of which is the sum of the scores for the internalizing problems and the externalizing problems); other problems (which include eating disorders, sleep disorders, sphincter control, physical problems, and sluggish cognitive time); and DSM-IV-oriented scales (anxiety problems, somatic problems, attention deficit hyperactivity disorder, opposition and challenging problems, and conduct problems).

The weighted results of the CBCL/6-18 allow for the classification of children and adolescents into normal, borderline, or clinical ranges. On the social scales, the scores are considered clinical when they are under 30, borderline when they are between 30 and 33, and normal when they are over 33. On the behavior scales, the scores are considered clinical when they are over 70, borderline when they are between 67 and 70, and normal when they are under 67. When the total score is calculated, clinical scores are defined as those over 63, while borderline scores are those between 60 and 63, and normal scores are those under 60.

The genomic DNA samples were obtained from 8 mL of total peripheral blood collected in tubes with EDTA (0.6 M), pH 8.0, as anticoagulant. Genomic DNA was purified from peripheral leukocytes according to standard protocols by lysing with proteinase K (Boehringer Mannhein, Germany), extracting with phenol/chloroform, and precipitating with ethanol (23 [Bibr B24]). To determine genotypes for the rs6318 (C/G) SNP in the HTR2C gene, real-time PCR using TaqMan¯ allelic discrimination assay (Applied Biosystems, USA) and primers available from SNP Genotyping Assay (Applied Biosystems) were used. The total volume for all reactions was 7 µL containing TaqMan Genotyping PCR Master Mix 2X (3.5 µL), SNP Genotyping Assay 40X (0.175 µL), MiliQ water (2.325 µL), and 10 ng of each genomic DNA (1 µL). Reactions were performed in 96-well optical plates (0.1 ml MicroAmp, Applied Biosystems) and submitted to the following temperatures and cycles: a first cycle of 10 min denaturation at 95°C followed by 40 cycles of 15 s at 95°C and 1 min extension at 60°C. Amplification reactions were performed in a 7500 Fast Real-Time PCR System (Applied Biosystems). Validation studies demonstrate that the Applied Biosystems 7500 System SDS is a robust, reliable, and reproducible system for performing DNA quantification (24). Data were recorded and analyzed using 7500 System Sequence Detection Software^©^ (SDS, Life Technologies Corporation^©^, USA). All procedures were conducted at the Human Genetics Laboratory of the Department of Molecular Biology and Genetic Engineering of UNICAMP.

The minor allele frequency (MAF) was calculated by adding the total number of alleles and then determining the rate of the least frequent allele among the homozygotes, hemizygotes, and heterozygotes. The result was compared to the 1000 Genomes Project database (11).

Statistical analyses were performed in SPSS, version 22 (IBM, USA). The weighted CBCL results were arranged into two distinct groups with two categories each. The first group included individuals with no abnormalities (“normal”) versus those with any abnormalities (“borderline clinical range” plus “clinical range”). The second group was based on another paradigm: individuals with no or few abnormalities (“normal” plus “borderline clinical range”) versus individuals with more abnormalities (“clinical range”).

The chi-squared test and Fisher's exact test were applied to evaluate possible differences in the frequencies of C and G alleles from the rs6318 SNP relative to each subscale of each weighted grouped score, as described above, on the CBCL/6-18. The significance level adopted was 5%.

## Results

The study comprised 85 patients, 65 (76.5%) of whom were male. The mean age was 13.4±2.7 years (range 8 to 18 years old). Most of the caregivers who completed the CBCL/6-18 scale were women (84.7%), 50 (58.7%) of whom were the patients' biological mothers. Other respondents included fathers, grandparents, shelter caregivers, stepmothers, stepfathers, aunts, uncles, and older brothers or sisters.

Table 1 shows the genotype distributions, allele frequencies, and MAFs of the study subjects and from the global database.

**Table 1:** Genotype distribution of studied single nucleotide polymorphism (SNP) and minor allele frequency (MAF).

SNP	Genotype	Allele Frequency	MAF	MAF Database
rs6318	G/G and G 72.9% (10/52)	G = 76 (73.8%)		
	C/C and C 22.4% (4/15)	C = 27 (26.2%)	C = 26.2%	C = 17%
	C/G 4.7% (4)			

Table 2 shows the significant associations between the CBCL/6-18 results and the rs6318 SNP. The presence of the G allele was found to be associated with aggressive behavior and social problems, and aggressive behavior was found to be associated with heterozygosis in females.

**Table 2:** Associations between the CBCL/6-18 results and SNP rs6318.

	Presence of G (G, GG, GC)	Absence of G (C, CC)	X^2^	P value
**Entire Sample**				
Aggressive Behavior				
Normal+Borderline	34 (69.4)	15 (30.6)	4.547	0.033
Clinical	32 (88.9)	4 (11.1)		
Social Problems				
Normal	32 (68.1)	15 (31.9)	5.538	0.019
Borderline+Clinical	34 (89.5)	4 (10.5)		
	**Hemizygous Homozygous (C, CC, G, GG)**	**Heterozygous (CG)**		
**Entire Sample**				
Aggressive Behavior				
Normal+Borderline	49 (100)	0	5.713	0.029*
Clinical	32 (88.9)	4 (11.1)		
	**G**	**C**		
**Only Males**				
Aggressive Behavior				
Normal+Borderline	13 (31.7)	28 (68.3)	5.281	0.022
Clinical	2 (7.7)	24 (92.3)		

Data are reported as numbers and percentages. CBCL: Child Behavior Checklist; SNP: single nucleotide polymorphism.

* Fisher’s exact test.

There was no significant association for any of the other tested CBCL grouped scores (P>0.05) with the genotype of the rs6318 SNP of the *HTR2C* gene.

## Discussion

The current study investigated the possible associations between the rs6318 SNP of the *HTR2C* gene and the categories addressed on the CBCL/6-18 scale. The results suggest that associations between the SNPs studied and symptoms suggestive of externalizing disorders are frequent. Treatment with antipsychotics is a therapeutic option for these symptoms, which include difficulty controlling aggressive behavior, antisocial behavior, and impulse control (25–27).

Other associations between behavioral changes and the rs6318 SNP have been described in the literature. According to Okada et al. (28), the C allele is much more active than the G allele. Brummett et al. (29) found the same baseline cortisol levels in subjects with either C or G alleles. However, when exposed to emotion-inducing stimuli, such as stress, anger, and sadness, subjects with the G allele had significantly lower cortisol activation (P<0.001), less anger (P=0.08), and a less depressive mood (P=0.006). The SNPs were also found to be associated with norepinephrine turnover, which was most highly correlated with the rs6318 SNP (30–33 [Bibr B34]). The altered expression of the *HTR2C* gene may therefore be associated with increased operation of the hypothalamic-pituitary-adrenal axis and with greater amygdala reactivity (34,14).

When the rs6318 SNP was evaluated, aggressive behavior was found to be associated with the presence of the G allele (P=0.029) across the sample. When the sample was separated by sex, the association of aggressive behavior remained among hemizygote males with the G allele (P=0.022). In psychiatry, aggressive behavior is described as being strongly associated with social problems (14). Our findings corroborate this information, as associations between social problems and the presence of G allele were found both in females alone (P=0.045) and with both sexes considered together (P=0.019).

Other studies have reported the expected association between the rs6318 SNP and depressive or anxiety symptoms, but it was not found in this study (35–39 [Bibr B40]). A possible explanation could be a question of selection bias, as all of the study subjects were taking antipsychotic drugs due to their diagnoses involving severe externalizing behaviors. This fact may have led to the undervaluation of depressive and anxiety symptoms among the participants. It was noted, however, that though these individuals were brought to psychiatric consultations mainly because of externalizing problems, the co-occurrence of the CBCL/6-18 depressive syndrome and anxious syndrome categories were positive in 28 (32.9%) and in 26 (30.6%) individuals, respectively.

The fact that subjects were taking psychiatric drugs at the time of the CBCL/6-18 application was a limiting factor. Because the scale considers only the six months prior to its administration, the medication may have influenced behavioral symptoms and decreased parents' perceptions. Nevertheless, the results were statistically significant, suggesting that the symptoms were so intense that they were observable even when the children and adolescents were receiving pharmacological treatment.

According to the 1000 Genomes Project (11), the MAF is 17% for the 6318 SNP in the overall population. In the current study, the MAF was 26.2% for rs6318 SNP. According to the data available, however, this frequency varies widely between ethnic groups.

Racial mixing in Brazil is something that must always be taken into account in any genetic study involving the population as a whole. Brazil's multiracial population is the result of contact between native indigenous peoples, European settlers, enslaved Africans, and, more recently, immigrants from the period between the two world wars, which included people from Italy and Japan, among other ethnicities (40).

The associations found between the rs6318 single nucleotide polymorphism on the *HTR2C* gene and behavioral symptoms, especially aggressive behavior problems, aid in the identification of phenotypes associated with gene expression. However, longitudinal studies are needed to examine the possible causal relationship between polymorphisms and transient or durable psychiatric symptoms over the course of childhood and adolescent development. Epigenetics should also be considered in future studies. These characteristics and a genetic predisposition to mental illness should be considered when determining the best therapeutic approach for a given patient.
